# Comparative Neuroprotective Effects of Dietary Curcumin and Solid Lipid Curcumin Particles in Cultured Mouse Neuroblastoma Cells after Exposure to A*β*42

**DOI:** 10.1155/2017/4164872

**Published:** 2017-04-16

**Authors:** Panchanan Maiti, Gary L. Dunbar

**Affiliations:** ^1^Field Neurosciences Institute Laboratory for Restorative Neurology, Central Michigan University, Mt. Pleasant, MI 48859, USA; ^2^Program in Neuroscience, Central Michigan University, Mt. Pleasant, MI 48859, USA; ^3^Department of Psychology, Central Michigan University, Mt. Pleasant, MI 48859, USA; ^4^Field Neurosciences Institute, St. Mary's of Michigan, Saginaw, MI 48604, USA; ^5^Department of Biology, Saginaw Valley State University, Saginaw, MI 48604, USA

## Abstract

Aggregation of amyloid beta protein (A*β*) and phosphorylated tau (p-Tau) plays critical roles in pathogenesis of Alzheimer's disease (AD). As an antiamyloid natural polyphenol, curcumin (Cur) has a potential role in prevention of neurodegeneration in AD. However, due to limited absorption of the dietary Cur, the solid lipid Cur particles (SLCP) have been suggested as being more effective for AD therapy. In the present study, we compared the role of dietary Cur and SLCP on oxidative stress, neuronal death, p-Tau level, and certain cell survival markers in vitro, after exposure to A*β*42. Mouse neuroblastoma cells were exposed to A*β*42 for 24 h and incubated with or without dietary Cur and/or SLCP. Reactive oxygen species (ROS), apoptotic cell death, p-Tau, and tau kinase (including GSK-3*β* and cell survival markers, such as total Akt, phosphorylated Akt, and PSD95 levels) were investigated. SLCP showed greater permeability than dietary Cur in vitro, decreased ROS production, and prevented apoptotic death. In addition, SLCP also inhibited p-Tau formation and significantly decreased GSK-3*β* levels. Further, the cell survival markers, such as total Akt, p-Akt, and PSD95 levels, were more effectively maintained by SLCP than dietary Cur in A*β*42 exposed cells. Therefore, SLCP may provide greater neuroprotection than dietary Cur in Alzheimer's disease.

## 1. Introduction

Alzheimer's disease (AD) is an age-related, progressive neurological disorder characterized by memory impairment and neuropsychological disturbances [1, 2]. Accumulation of amyloid beta proteins (A*β*) and phosphorylated tau (p-tau) is the key pathological hallmarks of AD [1, 3, 4]. Neuroinflammation, oxidative stress, and failure in protein degradation pathways are associated with this complex disorder [1, 5, 6]. These processes decrease trophic support of neurons and contribute to the decline of cell survival mechanisms [7]. Therefore, the maintenance of cell survival proteins markers as well as decreasing in oxidative stress is critical factor for preventing or delaying neurodegeneration or neuronal loss. In addition, recent studiers revealed a strong link between environmental-induced epigenetic changes, which could be a risk factor for dementia in AD and other neurological diseases [8]. Although several attempts have been made to salvage degenerating neurons by using small molecules, drugs, and natural polyphenols, none of them have been able to prevent the loss of degenerating neurons in a significant manner. Some of the drugs have been used for symptom-based treatments, such as acetylcholine esterase inhibitors (e.g., tacrine, physostigmine, and velnacrine) [9] or glutamate receptor inhibitors (e.g., memantine) [10] and can provide temporary symptomatic relief, and some of them are partially effective in restoring normal cognition and reducing neurobehavioral abnormalities, but they often produce adverse side effects and cannot completely halt or cure the disease progression.

Recently, curcumin has been targeted for AD therapy, because of its pleiotropic actions, including antiamyloid [11, 12], antioxidant [13], and anti-inflammatory properties [14–17]. It is a natural polyphenol, derived from the root of the herb,* Curcuma longa* [18]. Unfortunately, the poor solubility, instability in physiological fluids, and low bioavailability of Cur are the major obstacles for achieving its optimum theranostic values [19, 20]. Because of its hydrophobic nature, several investigators have formulated the Cur with lipid particles. Recently, we began using a solid lipid Cur particle (SLCP) formula, which has been shown to increase its solubility, stability, and bioavailability, in vitro and in vivo [15, 21–23].

Given this, the present study was designed to compare the neuroprotective effects of dietary Cur and/or SLCP, in vitro, after exposure to A*β*42. Our results suggest that SLCP has greater neuroprotective effects compared to dietary Cur in terms of restoring several cell survival proteins and decreasing neuronal death, in vitro, after exposure to A*β*2.

## 2. Materials and Methods

### 2.1. Chemicals

A*β* peptide, Cur (~80% pure), HFIP [1,1,1,3,3,3-hexafluoroisopropanol, 3-(4,5-dimethylthiazol-2-yl)-2,5-diphenyltetrazolium bromide (MTT)], and other accessory chemicals were purchased from Sigma (St. Louis, MO). The solid lipid Cur particles (SLCP™) or Longvida® or Longvida® Optimized Curcumin contains 26% of Cur and was a kind gift from Verdure Science (Noblesville, IN). The SLCPs have been well characterized by us [23] and others [15, 17, 24]. Details' sources of all the antibodies are summarized in Table 1.

### 2.2. Peptide Preparation and Treatment

The A*β* peptide preparation for the study of neurotoxicity was described previously [25]. Briefly, synthesized A*β*42 was first dissolved in cold HFIP (1,1,1,3,3,3-hexafluoroisopropanol) to get disassembled-preformed aggregates and allow drying the A*β*42 overnight at room temperature, followed by nitrogen flow, and then stored at −20°C until subsequent use. Prior to experimentation, the peptide was dissolved in a 60 mM NaOH solution (final concentration: 6 mM), followed by dilution with fresh MEM to achieve the desired concentration, prior to being added to the cell culture dishes. Similar concentrations, of NaOH were also added to control cells.

### 2.3. Cell Culture

Mouse neuroblastoma cells (N2a, ATCC) were used for this study. Briefly, the N2a cells were grown with minimum essential medium (MEM, GIBCO) containing 10% heat-inactivated fetal bovine serum (FBS) and penicillin/streptomycin (1 *μ*g/ml). The culture was maintained at 37°C in a humidified atmosphere at 5% CO_2_. Prior to the experiment, the cells were grown either in 60 mm Petri dish or in glass coverslips with fresh MEM, lacking growth factors, depending on the experimental setup. For Cur and/or SLCP permeability studies, the primary hippocampal neurons were taken from mouse embryonic-16 (E16) pups and grown in neurobasal media, containing B27 supplementation for 7 days as described previously [25].

### 2.4. Curcumin and/or SLCP Treatment

Both Cur and SLCP dissolve more readily in methanol than other solvents, as described previously [26]. Therefore, the stocks of Cur and SLCP were dissolved in methanol and then diluted in the MEM (methanol was <1%) before being added to the Petri dish containing the cells. The permeability of Cur and/or SLCP at different time points in primary hippocampal and N2a cells was assessed using a fluorescence microscope (Leica, Germany).

### 2.5. Cell Viability by MTT Assay

To investigate which concentration and duration of A*β*42 exposure are more toxic and what concentration of Cur provides greater neuroprotection, we conducted a cell viability test, using a MTT [3-(4,5-dimethylthiazol-2-yl)-2,5-diphenyltetrazolium bromide] assay [25, 27]. Briefly, the N2a cells were grown in 96-well plates (COSTAR, Corning, USA) at a density of 3 × 10^5^ cells/ml. Cells were treated with freshly prepared concentrations of A*β*42 (in *μ*M: 1, 5, 10, 15, 20) at different time points (in hours: 3, 6, 12, 24, and 48). After standardization of neurotoxicity levels, 10 *μ*M of A*β*42 was used for all experiments with 24-h exposure and with treatment at different concentrations of Cur (in *μ*M: 10, 1, 0.1, and 0.01). Briefly, following treatments, 10 *μ*L of MTT (12 mM) was added to each well and incubated for 4 h at 37°C. Then, the stop solution was added and kept overnight at room temperature. The optical density was measured using a Synergy plate reader (Bio-TEK instruments, Winooski, VT). The results of three independent experiments (6 wells per condition) were normalized to the media control group and expressed as mean ± SD.

### 2.6. Detection of Reactive Oxygen Species (ROS)

The N2a cells were grown on coverslips and treated with A*β*42 in presence or absence of Cur and/or SLCP (1 *μ*M), as described previously. Following treatment, the medium was removed and the cells were washed three times with PBS. Membrane permeabilization was done with 0.5% Triton X-100 for 10 minutes at room temperature. Then the CellROX® Reagent (Molecular Probe) was added at a final concentration of 5 *μ*M and incubated for 30 min at 37°C. After incubation, the cells were washed with PBS thrice, 5 min each, and then counter-stained with propidium iodide, followed by three washes at 5 min each. The cells were observed under the fluorescent microscope (Leica, Germany), using appropriate filters (ex/em: 485/520). The green fluorescent signal indicated ROS level. Total fluorescent intensity of each field was measured using Image-J software (https://imagej.nih.gov/ij/) and at least thirty random fields were selected from three independent experiments to obtain a mean value.

### 2.7. Annexin-V Staining for Apoptotic Assay

The N2a cells were grown on coverslips, coated with poly D-lysine (0.1 mg/mL) in minimum essential medium (MEM, GIBCO) containing 10% heat-inactivated fetal bovine serum (FBS) and pen/strep (1 *μ*g/mL). Prior to the experiment, the media were replaced with fresh MEM, which lacked growth factors, and treated with A*β*42 (10 *μ*M), along with different doses of Cur and/or SLCP (in *μ*M: 1, 0.1, and 0.01), dissolved in methanol, and diluted with fresh MEM for 24 h. Then coverslips were washed in PBS twice, stained with Annexin-V-FITC (apoptosis detection kit; Biotium, Hayward, CA), as per manufacturer's instruction. The images were taken using a fluorescence microscope (Leica, Germany) with blue/green/red filters. The total number of cells and the numbers of apoptotic cells were counted per microscopic field and expressed as a percentage of dead neurons. At least 30 microscopic fields from three independent experimental setups were used for counting.

### 2.8. Immunocytochemistry of p-Tau

The N2a cells were cultured on poly-D-lysine as described above. After stipulated period of treatment, the coverslips were washed in PBS, twice, and then fixed with 4% paraformaldehyde for 15 min. Then the coverslips were washed with PBS thrice, 5 min each, and incubated with 0.5% Triton-X100 along with 0.3 M glycine, 1% BSA, and 10% normal goat serum (Santa Cruz Biotech, CA) for 1 h at room temperature. Then the coverslips were incubated with rabbit anti-p-tau monoclonal antibody (1 : 100; Cell Signaling Technology), which was dissolved in PBS, along with 10% goat serum, and placed on a shaker (120 rpm) at 4°C, overnight. On the next day, the cells were thoroughly washed with PBS, three times, for 10 min each. Then the cells were incubated with appropriate secondary antibodies (1 : 200) tagged with FITC (Molecular Probes, OR) for 30 min at room temperature, then washed thoroughly with distilled water, dehydrated, mounted on slides using aqueous antifading media (Sigma, St. Louis, MO), and visualized using a fluorescence microscope (Leica, Germany) with appropriate excitation and emission filters.

### 2.9. Western Blot

After the stipulated period in each experiment, the media were removed from the culture dish and washed thrice with sterile PBS (pH 7.4). The cells were scraped and lysed with cold radio immunoprecipitation assay (RIPA) buffer (10 mM Tris-Cl (pH 8.0), 1 mM EDTA, 0.5 mM EGTA, 1% Triton X-100, 0.1% sodium deoxycholate, 0.1% SDS, and 140 mM NaCl, pH 7.4) with protease and phosphatase inhibitors (Sigma) and sonicated (Fisher scientific) for 1 min in ice-cold conditions. Then the lysate was centrifuged at 16,000 ×g for 15 min at 4°C. The supernatant was collected and aliquoted in PCR tubes and stored at −80°C until use. Total protein concentrations for individual samples were determined using the Pierce BCA protein assay (Thermo Scientific, Rockford, IL). Samples were added with equal amount of 2x SDS-sample buffer (125 mM Tris-HCl, pH 6.8, 4% sodium dodecyl sulfate, 20% glycerol, and 10% 2-mercaptoethanol) and boiled for 2 min. Approximately 50 *μ*g of protein, per lane, was loaded and electrophoresed on 10% Tris-glycine gel and transferred to PVDF membrane (Millipore, Bedford, MA). After probing with respective primary and secondary antibodies (Table 1), the blots were developed with Immobilon™ Western Chemiluminescent HRP-substrate (Millipore, Billerica, MA). The relative optical density was measured using Image-J software (https://imagej.nih.gov/ij/). To ensure equal protein loading in each lane, the blots were stripped and reprobed for *β*-tubulin.

### 2.10. Statistical Analysis

The data were expressed as mean ± SEM. Data were analyzed using one-way analysis of variance (ANOVA), followed by post hoc Tukey HSD (honestly significant difference) test. Probability ≤ 0.05 was considered as statistically significant.

## 3. Results

### 3.1. Greater Cellular Permeability for SLCP Than Dietary Cur

**Figure 1:**
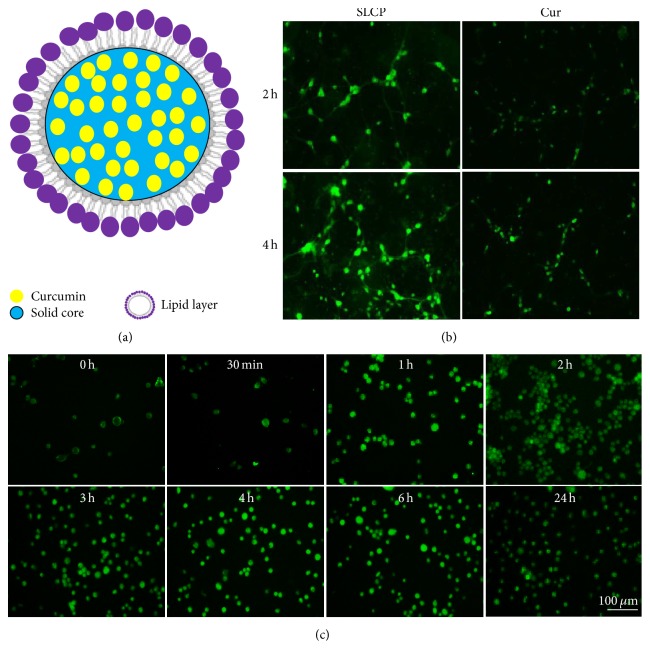
SLCP has greater cellular permeability than dietary Cur. (a) Schematic diagram showing the formulation of SLCP, indicating that Cur is coated with solid lipid core of a lipid bilayer. (b) Mouse embryonic primary hippocampal neurons (7 days old) were treated with Cur and SLCP in different time points, with SLCP showing greater permeability than dietary Cur after 2 and 4 h incubation. N2a cells were treated with SLCP (1 *μ*M) up to 24 h and maximum permeability was observed after 1 h with no significant change of permeability, even up to 24 h of incubation. Scale bar indicates 100 *μ*m and is applicable to all images.

To compare the cellular permeability of dietary Cur and SLCP, we treated the 7-day hippocampal neurons for 2 and 4 h. We found greater cellular permeability in the case of SLCP after 2 and 4 h of incubation, when compared to dietary Cur (Figure 1(b)). We also monitored the permeability of SLCP in N2a cells from 0 h to 24 h and observed that maximum permeability was reached within first hour of SLCP administration and intensity becomes stable up to 24 h (Figure 1(c)).

### 3.2. Curcumin Increases Cell Viability after Exposure to A*β*42

**Figure 2:**
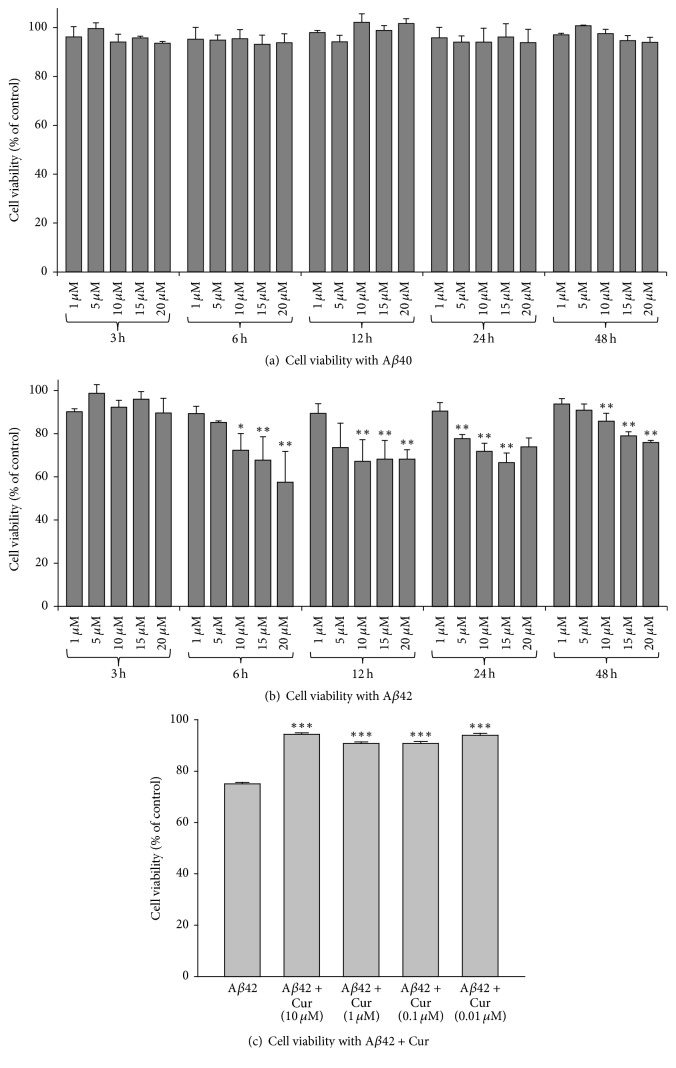
Cell viability after exposure to different concentrations of A*β*0 and A*β*42 at different time points and the protective effects of Cur. N2a cells were treated with different concentrations of A*β*40 and A*β*42 (*μ*M) at different time points, respectively. Data are expressed as mean ± SD. (a) No significant neurotoxicity was observed in the case of A*β*40 treated cells. (b) Cell viability tended to decrease with decreasing concentrations and increasing time with A*β*42 incubation. ^*∗*^*p* < 0.05; ^*∗∗*^*p* < 0.01 compared to control. (c) All concentrations of Cur prevented neuronal death following exposure to 10 *μ*M of A*β*42 for 24 h (^*∗∗∗*^*p* < 0.001 relative to A*β*42 alone).

To investigate dose and duration of A*β*42 toxicity, we treated the N2a cells with different concentrations of freshly prepared A*β*40 and A*β*42 (1, 5, 10, 15, and 20 *μ*M) which were incubated with different time points (3, 6, 12, 24, and 48 h). Although we did not observe a significant decrease of cell viability when they were treated with different concentrations of A*β*40 at different time points (Figure 2(a)), we did find a significant decrease of cell viability with 10, 15, and 20 *μ*M after 6–24 h of incubation with A*β*42 (Figure 2(b)). We choose A*β*42 based on these results and previous work with other A*β* peptides [25, 27, 28]. We choose the 10 *μ*M concentration of A*β*42 at 24 h, as this was minimum concentration with consistent neurotoxic effects [25, 27, 29].

After treating N2a cells with 10 *μ*M concentration of A*β*42 for 24 h and with different doses of dietary Cur (in *μ*M: 10, 1, 0.1, and 0.01); we observed that all the concentrations of Cur prevented A*β*42-induced cell death (Figure 2(c)).

### 3.3. SLCP Prevented ROS Formation

**Figure 3:**
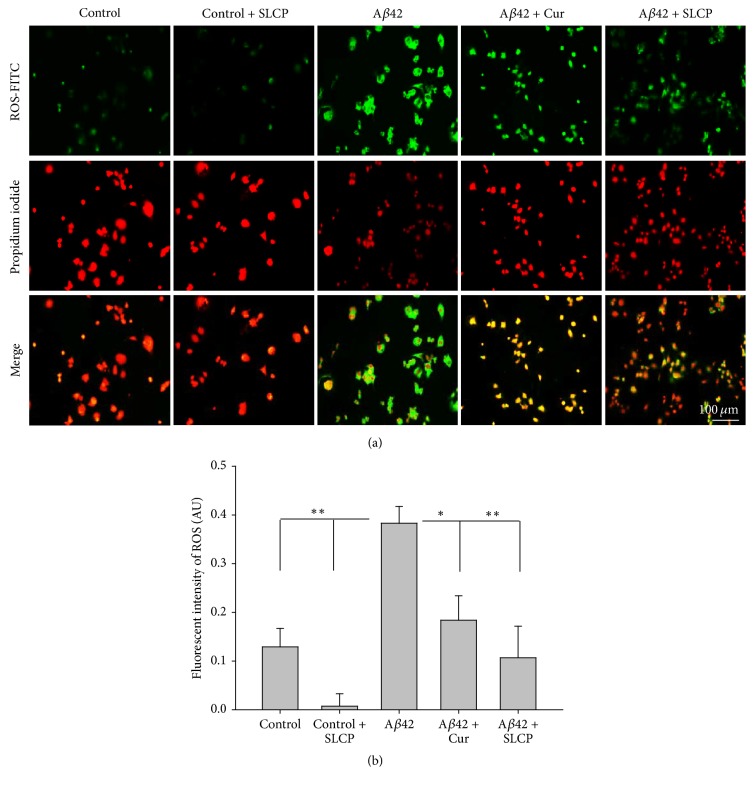
SLCP decreases A*β*42-induced ROS formation. N2a cells were treated with A*β*42 (10 *μ*M) for 24 h in presence or absence of SLCP (1 *μ*M) before being stained with CellROX oxidative stress reagents for detection of ROS levels. (a) ROS immunofluorescent (green) was increased by A*β*42, while Cur- and SLCP-treated cells decreased the green fluorescent signal. (b) Quantification of the fluorescent signals showed that A*β*42-treated cells had significantly increased (^*∗*^*p* < 0.05; ^*∗∗*^*p* < 0.01) ROS production, while SLCP prevented the A*β*42-induced increase in ROS more effectively than did dietary Cur. Scale bar indicates 100 *μ*m and is applicable to all other images.

To test whether low dose (*μ*M) of Cur and/or SLCP was able to rescue N2a cells from A*β*-induced ROS production and apoptotic death, the N2a cells were challenged with 10 *μ*M of A*β*42 and treated with SLCP (1 *μ*M) for 24 h. We observed that ROS levels were significantly increased (*p* < 0.01) by A*β*42 exposure, and they were reduced by SLCP (Figures 3(a) and 3(b)).

### 3.4. Greater Prevention of A*β*42-Induced Neuronal Apoptosis with SLCP Than Dietary Cur

**Figure 4:**
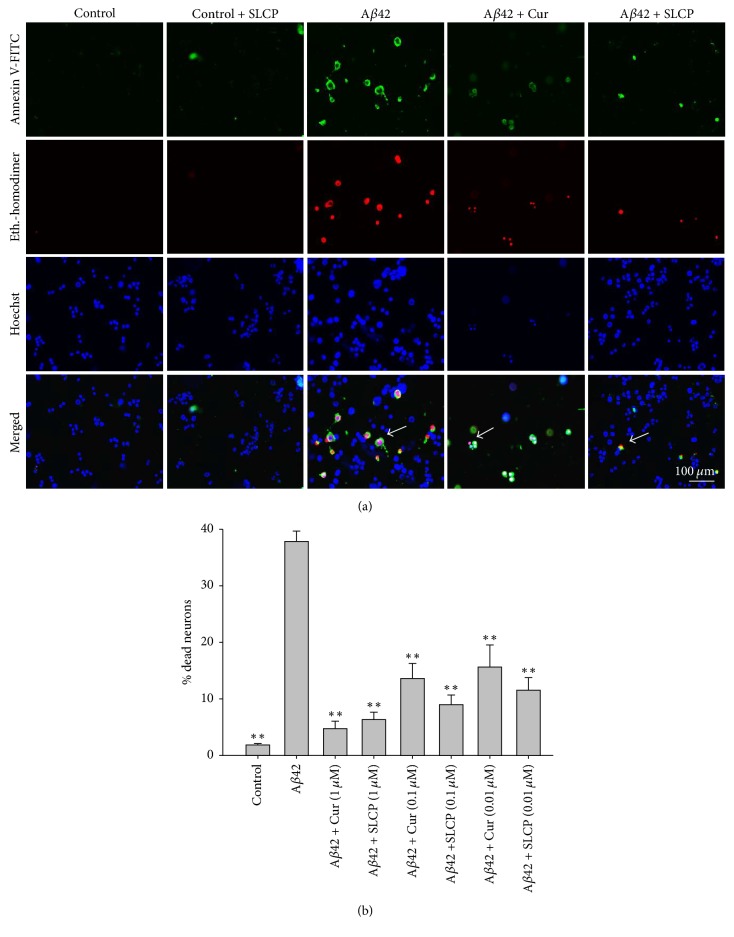
SLCP prevented A*β*42-induced neuronal apoptosis. N2a cells were treated with A*β*42 (10 *μ*M) for 24 h in presence and absence of dietary Cur and SLCP (1 *μ*M) and apoptotic cell death was monitored with Annexin-V staining. (a) Images showed that apoptotic death was increased after exposure to A*β*42, whereas SLCP treatment prevented the neuronal death. (b) Morphometric analysis indicates that A*β*42 induces almost 40% neuronal apoptosis and treatment of different concentrations of Cur and/or SLCP decreased cell death significantly (^*∗∗*^*p* < 0.01 compared with A*β*42 alone). Green fluorescent indicates apoptotic death; red is nuclear stain. Arrows indicate apoptotic cells. Scale bar indicates 100 *μ*m and is applicable to all other images.

Annexin-V staining was performed to monitor A*β*42-induced apoptotic death and to determine whether Cur and/or SLCP is protective. We found that A*β*42 (10 *μ*M) induced almost 40% neuronal apoptosis, whereas 75–85% of neurons were rescued from dying using 1 *μ*M concentrations of Cur (87%) and SLCP (83%). Similarly, 65–75% reduction in neuronal death was observed with 100 nM Cur (64%) and SLCP (76%) and by 50–70% using 10 nM Cur (58%) and SLCP (69%) concentrations (Figures 4(a) and 4(b)).

### 3.5. Cell Death and Cell Survival Markers after Exposure to A*β*42 and Treatment with Cur and/or SLCP

**Figure 5:**
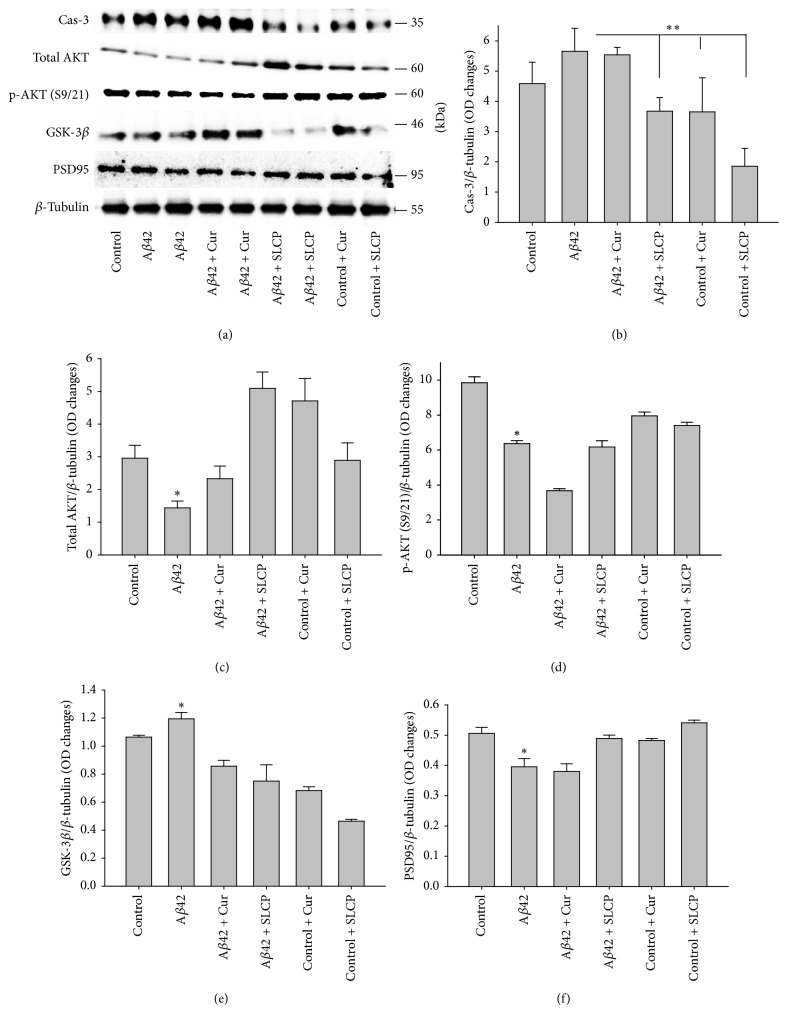
Cell survival markers proteins were enhanced by dietary Cur and/or SLCP after exposure to A*β*42. N2a cells were treated with A*β*42 (10 *μ*M) for 24 h in absence or presence of dietary Cur and SLCP (1 *μ*M). Western blot data indicated a significant increase in Cas-3 (b) and GSK-3*β* (e), with decreases in total Akt level (c), p-Akt (c and d), and PSD9 (f), whereas Cur and/or SLCP ameliorated these effects. ^*∗∗*^*p* < 0.01 and ^*∗*^*p* < 0.05 in comparison to group treated only with A*β*42.

We investigated different cell death and cell survival markers by Western blot analyses after exposure of A*β*42 and after treatment with Cur and/or SLCP (1 *μ*M). We observed that caspase-3 (Figures 5(a) and 5(b)) and GSK-3*β* (Figures 5(a) and 5(e)) were significantly increased (*p* < 0.05) with A*β*42-treatment, whereas SLCP, but not Cur, was able to decrease these protein levels. In contrast, exposure to A*β*42 downregulated total Akt (Figures 5(a) and 5(c)), p-Akt (Figures 5(a) and 5(d)), and PSD95 (Figures 5(a) and 5(f)), whereas Cur and/or SLCP treatments restored their levels (*p* < 0.05).

### 3.6. SLCP Decreased Phosphorylated Tau after Exposure to A*β*42

**Figure 6:**
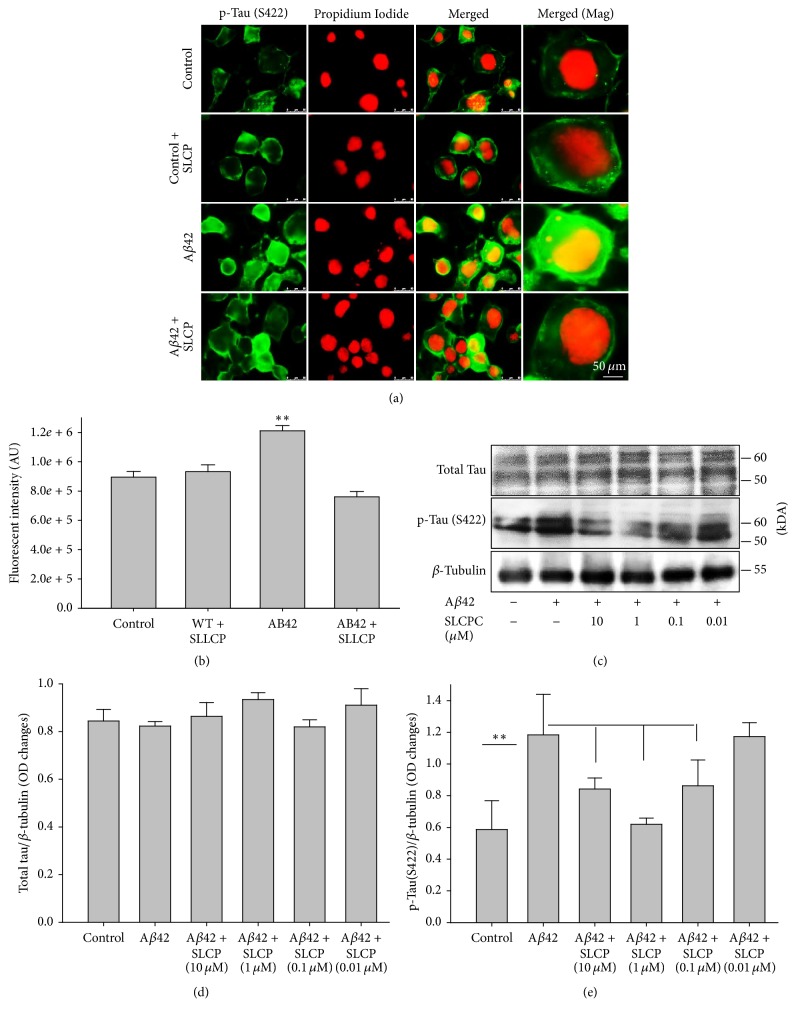
A*β*42-induced p-tau was decreased by SLCP treatment. (a-b) N2a cells were treated with A*β*42 (10 *μ*M) for 24 h in absence or presence of different concentrations of SLCP. The p-tau immunofluorescent signal was significantly increased after A*β*42 exposure (^*∗∗*^*p* < 0.01 relative to control) and normalized by SLCP (1 *μ*M) treatment. Scale bar indicates 50 *μ*m and is applicable to all other images. (c–e) Western blot data showed that p-tau (S422) was significantly increased by A*β*42 exposure, whereas SLCP treatment protected against such increase at 10, 1, and 0.1 *μ*M levels (^*∗∗*^*p* < 0.01, in comparison to all other groups with different concentrations of SLCP treatment, except those given 0.01 *μ*M).

Accumulation of p-tau as a marker for neurofibrillary tangles in the intracellular spaces is another pathological hallmark of AD. Our immunofluorescent study showed that exposure to A*β*42 significantly increased (*p* < 0.01) p-tau (S416), without alterations of total tau, whereas SLCP treatment prevented this increase (Figure 6(a)). However, when we treated N2a cells with different concentrations of SLCP (10 *μ*M, 1 *μ*M, and 0.1 *μ*M) after exposure to A*β*42, our Western blot data showed that 10 *μ*M, 1 *μ*M, and 0.1 *μ*M of SLCP were able to decrease p-tau significantly, but the 0.01 *μ*M concentration did not (Figures 6(b)–6(d)) (*p* < 0.01).

## 4. Discussion

The primary goal of this study was to compare the neuroprotective capabilities of the dietary Cur with those of SLCP after A*β*42 exposure in vitro. To this end, we have investigated the levels of ROS, neuronal death, and p-Tau as well as cell survival and cell-death-related protein markers in mouse neuroblastoma (N2a) exposed to A*β*42. We observed that the A*β*-induced cell death was prevented by dietary Cur and/or SLCP treatment, along with improvement of cell survival markers.

Cur has been suggested as a potential treatment of AD during the past few years, but its poor absorption and rapid degradation of most of our body fluids have limited its clinical utility. To circumvent this, we compared a new formulation of Cur that has been loaded with lipid particles (SLCP). In this study, we used primary hippocampal neurons and treated them with both dietary Cur and SLCP and observed that SLCP is usually more permeable than dietary Cur (Figure 1(b)). This is due to its being coated with a lipid bilayer, which can facilitate permeability through the membrane lipid layer [26].

To confirm whether Cur or SLCP can rescue A*β*42-induced cell death, we performed an MTT assay [30]. Initially, we did a dose- and duration-dependent study of cell viability after A*β*42 exposure. Although the cell death continued at longer time points with higher doses, we determined that 10 *μ*M concentration and 24 h incubation times were optimal [25, 27] for investigating neuroprotective effects of Cur and/or SLCP. When we treated the cells with Cur, we observed that cell survivability was increased by 50–60% (Figure 2). Similarly, we also performed Annexin-V staining, which reflects apoptotic death, and we found that Cur and/or SLCP treatment reduced cell death produced by A*β*42 exposure (Figure 4). We have performed most of these experiments with 1 *μ*M of Cur and/or SLCP, but we were also interested in whether lower doses, such as nanomolar (nM) concentrations, can inhibit the A*β*2-induced neuronal apoptosis. To answer this question, we treated N2a cells with different doses (in *μ*M: 1, 0.1, and 0.01) of Cur and/or SLCP. We observed that all doses of Cur and/or SLCP were able to prevent A*β*42-induced apoptosis in a dose-dependent fashion (Figure 4), suggesting nM concentration of Cur is sufficient to rescue cell death from A*β*42 insult.

One of the causes of neuronal death after A*β*42 exposure is an increase in oxidative stress, leading to elevated levels of ROS. When we labeled the cells with CellROX oxidative stress reagents, we found that there was significant increase in the amount of ROS production in A*β*42-exposed neurons as indicated by an elevated green fluorescent signal (Figure 3). Cur and/or SLCP treatments were able to decrease the ROS production. Cur is a potent antioxidant, which can scavenge most of the ROS and reactive nitrogen species (RNS), thus decreasing oxidative stress [13]. In addition to its effects in reducing ROS, the inhibition of neuronal death by Cur and/or SLCP treatment after A*β*42 exposure may be accomplished by decreasing caspase-3 levels. This hypothesis is supported by our findings that caspase-3 is involved in apoptotic death, which was decreased by SLCP, but not by Cur treatment. Our data is consistent with that of Ray and colleagues who have used a water-soluble polymeric nanoparticle encapsulated curcumin (NanoCurc™) and they showed that this Cur formula protects from ROS (H_2_O_2_)-induced oxidative damage in human neuroblastoma cells (SK-N-SH) [31, 32]. Similarly, when they injected this Cur formula in athymic mice, intraperitoneally (IP) at a dose of 25 mg/kg body weight twice daily, they found a decreased levels of H_2_O_2_ as well as inhibition of caspase-3 and caspase-7 activities in the brain, accompanied by increased antioxidant levels, such as reduced glutathione (GSH) levels [31]. Although we did not measure the intracellular Cur level in this study, when dietary Cur or SLCP is injected to 5xFAD mice, we have observed that significant amounts of free Cur reach the brain and bind to amyloid plaques [26]. Similarly, in our previous work, we observed neuroprotective effects when we fed the 3xTg rat, 5xFAD mice, and CAG140-knock-in Huntington's disease mice with SLCP formula (555 ppm for 3 months) and measured the Cur level in their brain tissues by HPLC-MS/MS spectroscopy; we found ~250–300 nM/g tissue [15, 17, 33]. This suggests that these special Cur formulas might be more effective in rescuing A*β*42-exposed neuronal death, relative to dietary Cur.

Interestingly, we observed that p-Tau was increased after A*β*42 exposure [34] without alteration of total tau, and we observed that SLCP also decreased p-Tau level as seen by immunofluorescence, as well Western blot data (Figure 6). The p-Tau is one of the hallmark pathologies in AD, which causes formation neurofibrillary tangles (NFTs). Decreased p-tau levels by SLCP treatment may be due to inhibition of tau kinases, such as GSK-3*β* [35, 36], which were increased by A*β*42 treatment and which have been observed in the AD brain [37–39]. Why the dietary Cur was not able to diminish the GSK-3*β* levels is not clear to us, but we speculate that more Cur was delivered by SLCP than by dietary Cur. However, we also found that treatments with SLCP restored the protein degradation system, such as molecular chaperones and autophagy lysosomal pathways, which are critical for misfolded protein degradation, including p-tau and A*β* [40, 41].

Cur and/or SLCP treatments protect the neurons, not only through inhibition of ROS or by decreasing Cas-3 level, but also by activating Akt and p-Akt levels [42, 43]. Akt is one of the cell survival proteins, which becomes downregulated in different stress conditions, included in several neurodegenerative diseases [44]. Therefore, cell survival pathways might also play important roles in the restoration of protein clearance pathways, such as HSPs and ALP, and provide overall neuroprotection following Cur or SLCP treatments [45]. Of course, there are several other ways where Cur may provide neuroprotection, including increased secretion of several growth factors, including BDNF, NGF, and IGF [46, 47]. Although we did not investigate changes in these neurotropic factors in the present study, we have found that Cur restores BDNF in other animal models of neurodegenerative diseases, which is under investigation. These growth factors may help protect neurons from dying by A*β* exposure.

Alzheimer's disease is a multifactorial disease, which involves accumulation of misfolding amyloid protein [1], neuroinflammation, oxidative stress, involvement of environment-induced epigenetic changes, such as DNA oxidation or methylation [8, 48, 49], and alteration of histone structure [50]. Recently, epigenetic evidence suggests that dementia or memory loss is a gradual deterioration of crucial cellular pathways, which cause neurodegeneration and dysfunction, whereas manipulation of epigenetic mechanisms could be used to assist in the detection, prevention, and reversal of such processes before the onset of clinical dementia [8]. Therefore, multiple approaches, such as use of antiamyloid, antioxidant, and anti-inflammatory agents, as well as epigenetic manipulations, could provide better theranostic value to address complex diseases, like AD [51]. 

*Conclusions.* Taken together, Cur can prevent A*β*42-induced neuronal death by inhibiting ROS production or through blocking apoptotic pathways and boosting cell survival pathways. Because of its greater permeability or stability, SLCP conferred more neuroprotection than dietary Cur. Given that Cur is a promising compound for AD therapy, our findings help provide new insights into understanding the neuroprotective mechanisms involved in preventing the types of neurodegeneration observed in AD.

## Figures and Tables

**Table 1:** Sources of different antibodies used in this study.

Antibodies	Source	Type	Company	Catalog numer	Address
Total tau	Rabbit	Polyclonal	Santa Cruz Biotech	sc-5587	Santa Cruz, CA
p-Tau (S416)	Rabbit	Monoclonal	Cell signaling Technology	15013	Danvers, MA
Akt	Rabbit	Monoclonal	Cell signaling Technology	4685	Danvers, MA
pAkt (Ser473)	Rabbit	Monoclonal	Cell signaling Technology	9271	Danvers, MA
GSK-3*β*	Rabbit	Polyclonal	Cell signaling Technology	12456	Danvers, MA
p-GSK-3*β* (Ser9)	Rabbit	Monoclonal	Cell signaling Technology	9323	Danvers, MA
PSD95	Mouse	Monoclonal	Santa Cruz Biotech	Sc-32290	Santa Cruz, CA
Caspase-3	Rabbit	Polyclonal	Abcam	Ab13847	Cambridge, MA
*β*-Tubulin	Rabbit	Monoclonal	Cell Signaling Technology	2128	Danvers, MA

## Abbreviations

Cur: Curcumin
SLCP: Solid lipid curcumin particles
AD: Alzheimer's disease
A*β*: Amyloid beta protein
ANOVA: One-way analysis of variance
AU: Arbitrary unit
HSD: Honestly significant difference
DMEM: Dulbecco modified Eagle's medium
EDTA: Ethylene diamino tetra acetic acid
EGTA: Ethylene glycol tetra acetic acid
FBS: Fetal bovine serum
MTT: 3-(4,5-Dimethylthiazol-2-yl)-2,5-diphenyltetrazolium bromide
OD: Optical density
SDS: PAGE-sodium dodecyl sulfate polyacrylamide gel electrophoresis
TBS: Tris buffer saline
RIPA: Radio immunoprecipitation assay
Cas: Caspase.
